# Complement tumor-crosstalk: the role of C5a receptors in urothelial carcinomas

**DOI:** 10.3389/fimmu.2026.1885439

**Published:** 2026-09-07

**Authors:** Lea Sophie Lütje, Caroline Gruner, Christian Marcel Karsten, Axel Stuart Merseburger, Marie Christine Roesch

**Affiliations:** 1 Department of Urology, University Hospital Schleswig-Holstein Campus Lübeck, Lübeck, Germany; 2 Institute for Systemic Inflammation Research, University of Lübeck, Lübeck, Germany

**Keywords:** anaphylatoxin C5a, C5aR1, C5aR2, cancer, complement, therapy, urothelial carcinoma

## Abstract

Urothelial carcinoma, the second most common urological malignancy, can arise in both the lower and upper urinary tract, with approximately 90–95% arising in the bladder, while urothelial carcinomas of the upper urinary tract account for only 5–10%. Treatment options depend on many factors, such as grade and risk factors, but include surgery, chemotherapy, and immunotherapy. Due to the unique pathological and molecular characteristics of each patient, some therapies are ineffective, and the need for targeted therapies and precision medicine is growing. The interactions of malignant cells within the tumor microenvironment can be decisive for the course of therapy. These interactions include components of the complement system, which is a highly conserved part of the innate immune system. The role of the complement system, especially the anaphylatoxin receptors C5aR1 and C5aR2, in cancer is complex. C5aR1 appears to be tumor-promoting by inhibiting the T cell response and modulating the TME, while C5aR2 plays an ambivalent role and was shown to counteract tumor development in some mouse models while in other types of cancer it stimulated tumor growth and led to chemoresistance. In urothelial carcinomas, C5aR1 has been described as a prognostic factor, while C5aR2 has not been considered to date. Here, we briefly summarize our current knowledge of the complex functions of C5a receptors in cancer. In particular, this is the first focused summary of data on C5a receptors in urothelial carcinomas.

## Introduction

In developed countries urothelial carcinoma (UC) ranks as the second most prevalent urological malignancy (1). This neoplasm can develop in both the lower urinary tract (comprising the bladder and urethra) and the upper urinary tract (including the renal pelvis and ureter). The vast majority of UC cases, approximately 90 – 95%, originate in the bladder (bladder cancer, BC), whereas the upper tract urothelial carcinomas (UTUC) comprise only 5- 10% of all UC diagnoses (2). BC ranks as the seventh most frequently diagnosed malignancy among males worldwide and the tenth taken both sexes into account (3). At initial diagnosis, approximately three-quarters of patients with BC present with non–muscle-invasive disease (4), who tend to have a relatively favorable prognosis, compared to those with muscle-invasive or more advanced disease stages (3, 5). Treatment options depend on whether the patient presents with a non-muscle invasive BC (NMIBC) or a locally advanced or metastatic disease. A detailed summary of the current treatment options has already been published elsewhere (6).

The incidence of UTUC peaks in individuals aged between 70 and 90 years, with the disease occurring approximately twice as frequently in males as in females (7). At the time of diagnosis, nearly two-thirds of UTUC patients present with muscle-invasive disease (8). Treatment regimens for UTUC are summarized in the “European Association of Urology Guidelines on Upper Urinary Tract Urothelial Carcinoma” (9).

Patients exhibit unique pathological and molecular characteristics, even with the same diagnosis (6), which means that “one-size-fits-all” therapies do not prove effective for everyone. To improve patient care in the future and offer personalized therapies, understanding the complex network of malignant cell within the tumor microenvironment (TME) is important, as this is where interactions between the cancer cell and soluble mediators take place, among which are complement components (10).

The complement system (CS) is a complex multifunctional system that comprises more than 40 plasma and membrane proteins, which contribute to its role in innate and adaptive immunity (11). The mechanisms of complement activation, canonical and non-canonical, have been extensively reviewed in the literature (10, 12–21). In cancer the CS has been shown to have a paradoxical role by acting as a tumor suppressor as well as a promotor (10). While robust activation can mediate tumor surveillance by inducing lysis, immunogenic cell death (ICD) (22), antigen presentation (23) and adaptive immune activation (24), most tumors cause chronic, sublytic complement activation (25). This persistence is often due to the overexpression of complement regulatory proteins (CRPs) on tumor cells, which prevents direct cytotoxic killing via the Membrane Attack Complex (MAC). Instead, this sustained activation generates complement activation fragments such as C3a, C5a, C5b (25). These fragments among other mechanisms then drive chronic inflammation, recruit immunosuppressive myeloid cells and polarize tumor-associated macrophages (TAMs) toward a pro-tumorigenic phenotype (26–28). Consequently, rather than killing cancer cells, complement signaling aids immune evasion (29).

Although several components of the complement system play a role in cancer biology, several studies have highlighted the important role that C5a/C5aR signaling plays in tumor immunity and point to this signaling pathway as a potential therapeutic target; for this reason, C5aR1 and C5aR2 were chosen as the focus of this review. Our understanding of the role of C5aR1 and C5aR2 in various types of cancer has improved in the last decade, however, we are still in the early stages of determining the role of these receptors in the development and progression specifically of urothelial carcinomas, as well as possible therapeutic approaches. Here, we present an overview of our current state of knowledge and possible future developments on this topic. In comparison to existing reviews by, for instance, Merle et al., Borisov et al. and Garlanda et al. (10, 21, 30) this mini-review focuses specifically on the role of C5a receptors in UCs and integrates the latest data on C5aR1/C5aR2 signaling, pan-cancer analyses, and UC-specific proteomics.

### The known roles of the C5a/C5aR1 and C5a/C5aR2 axes in cancer

It was long assumed that the CS could eliminate tumor cells in the same way as bacteria, but the work of Markiewski et al. in 2008 provided the first evidence that the CS has an ambivalent function in cancer. The authors were able to show that the host is negatively affected by the activation of C5aR1. Using a subcutaneous cancer model with highly tumorigenic lung epithelial cells, it was reported that myeloid suppressor cells (MDSCs) are recruited to the tumor core *via* C5aR1, which in turn resulted in a limited CD8^+^ T-cell response (31). A mouse model for metastatic breast cancer later confirmed these findings, as it was able to show how C5aR1 promotes lung metastasis by influencing the immunological microenvironment in the metastatic niche, affecting transforming growth factor beta (TGF-β) and interleukin-10 (IL-10) production by immature myeloid cells, which leads to the inhibition of anti-tumor T cell responses in the lung (28). In line with that, a suppressed T-cell response due to C5aR1 activation on neutrophils led to the progression of ovarian cancer (32, 33). Synergistic C5a/PD1 blockade, in contrast to either treatment alone, protects against the progression and metastasis of Non-Small Cell Lung Cancer (NSCLC) in humans by limiting the abundance of MDSCs and reversing CD8^+^ T cell exhaustion. These findings further support the effect of C5aR1 on the TME (34). Furthermore, the crucial role of the C5a/C5aR1 axis in attracting MDSCs and suppressing effector T cell responses was confirmed in several other mouse models, including hepatic metastases (35), lung cancer (36) and melanoma (37) Additionally, it was shown that lung cancer metastasis is promoted by C5a through the amplification of tumor-supporting activity of neutrophil MDSCs *via* the formation of neutrophil extracellular traps (NETs). In a mouse model of lung metastasis, a combined inhibition of the C5a/C5aR1 axis and NETosis led to a reduction in metastasis (38).

On the other hand, the role of C5aR2 in cancer has not yet been elucidated, as there is only little and controversial research available (39). In several murine models including melanoma (37), colorectal cancer (40), and cutaneous squamous cell carcinoma (41) C5aR2 has been shown to restrict tumor growth by counteracting the immunosuppressive effects mediated by C5aR1 signaling. However, in human disease settings, the association is often detrimental. In glioma patients, increased C5aR2 expression correlates with poor prognosis and the co-expression of multiple immune checkpoints such as PD-L1, PD-1, cytotoxic T-lymphocyte-associated protein 4 (CTLA-4), and T-cell immunoglobulin and mucin-domain containing 3 (TIM-3) alongside markers of tumor-associated macrophages (TAMs), type 2 helper (Th2) and regulatory T (Treg) cells (42). Similarly, in breast cancer, high C5aR2 expression was related to poor prognosis and was shown to be involved in immune cell infiltration as well as malignant characteristics (39). Mechanistic insights reveal a complex interplay within the TME. For instance, in breast and lung cancer, specific carcinoma-associated fibroblasts (CAFs) defined by the expression of CD10 and C5aR2 have been identified. These cells are implicated in chemoresistance and poor survival rates by sustaining nuclear factor kappa-light-chain-enhancer of activated B-cells (NF-κB) activation through complement signaling via C5aR2, creating a protective niche for cancer stem cells (CSCs) with high levels of IL-6 and IL-8, which are important for CSC survival and maintaining cancer cell stemness. The efficacy of this function is highlighted by the successful intervention using an anti-C5aR2 antibody, which halts tumor formation and restores chemosensitivity (43). Based on these studies C5aR2 should be viewed as a context-depended regulator rather than a receptor with uniformly tumor-promoting or tumor-suppressing function. Its biological effect seems to depend on the tumor type, the cellular composition of the TME, and the prevailing inflammatory context. The apparent variability likely reflects its expression on both tumor cells and immune cells, as well as its atypical signaling properties and functional interaction with C5aR1. Taken together, these findings suggest that C5aR2 does not act as a fixed cancer-associated factor, but rather as a receptor whose functional profile is shaped by tumor-specific and microenvironment-dependent conditions.

**Figure 1:**
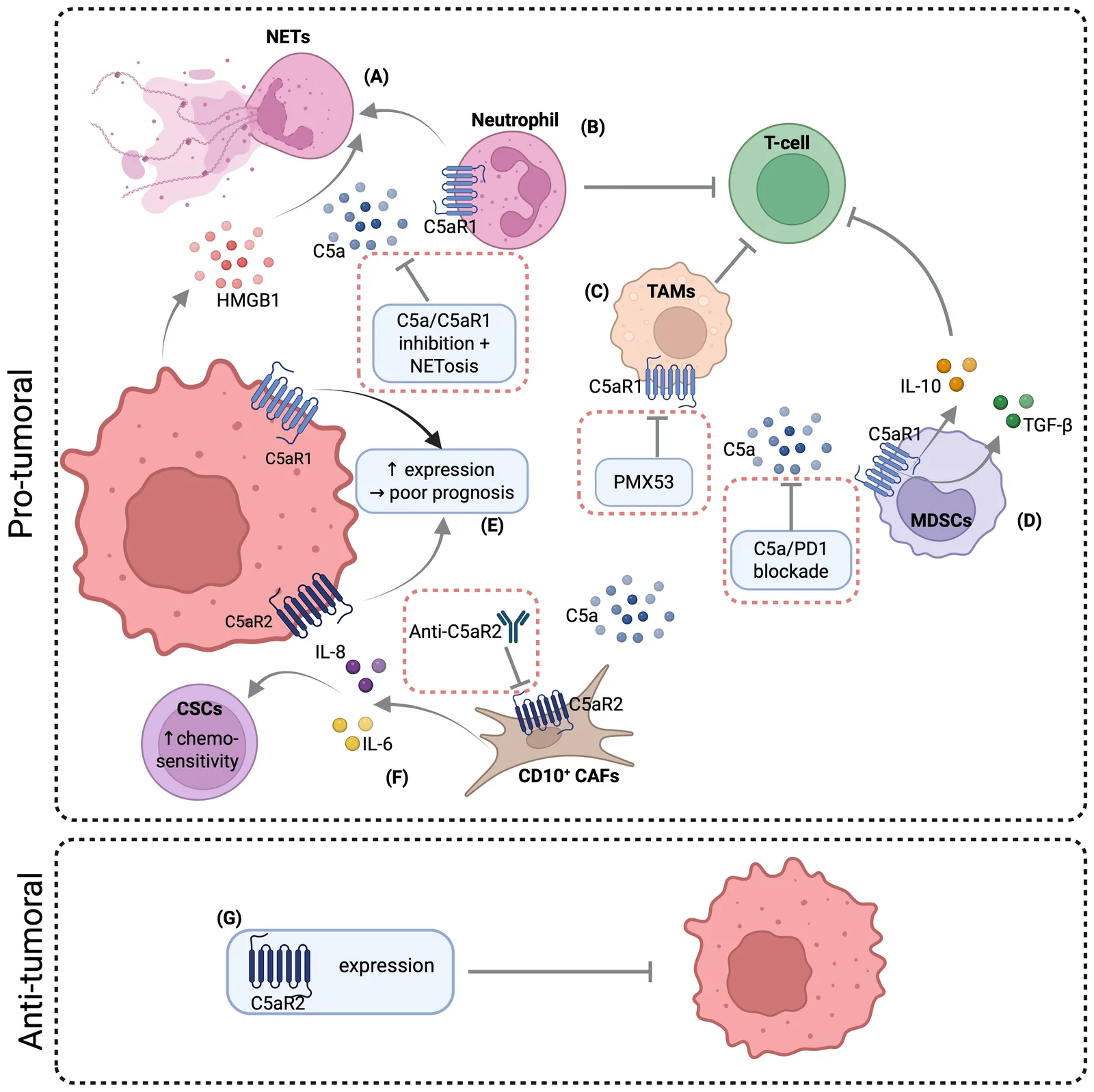
Schematic overview of the known roles of the C5a/C5aR1 and C5a/C5aR2 axes in cancer. **(A)** Metastasis is promoted by C5a through the amplification of tumor-supporting activity of neutrophils *via* the formation of neutrophil extracellular traps (NETs), which is mediated by the nuclear protein high mobility group box 1 (HMGB1) released from cancer cells. A combined inhibition of the C5a/C5aR1 axis and NETosis leads to a reduction in metastasis. **(B)** C5aR1 activation in neutrophils results in a suppressed T cell response. **(C)** C5aR1^+^ TAMs exhibit an immunosuppressive property, which contributes to an impaired CD8^+^ T cell function. **(D)** Recruitment of MDSCs to the tumor core *via* C5aR1. The activation of MDSCs leads to the release of the cytokines IL-10 and TGF-β which lower the CD8^+^ T cell response. C5a/PD1 blockade limits the abundance of MDSCs and reverses CD8^+^ T cell exhaustion. **(E)** High C5aR1 as well as high C5aR2 expression in tumor cells is related to poor prognosis. **(F)** CD10^+^C5aR2^+^ carcinoma-associated fibroblasts (CAFs) provide a constant source of IL-6 and IL-8 through complement signaling *via* C5aR2, thereby supporting cancer stem cells (CSCs), resulting in promotion of tumor growth and chemoresistance. The use of an anti-C5aR2 antibody effectively stops tumor formation and restores chemosensitivity. **(G)** Tumor growth-restricting effects of C5aR2 expression are found in mouse models for colorectal cancer, melanoma and cutaneous squamous cell carcinoma. Experimentally proven mechanisms specific to urothelial carcinoma (UC) are shown with black arrows while proposed mechanisms specific to UC are shown with grey arrows. Created in BioRender. Lütje, L (2026). https://BioRender.com/.

C5a/C5aR signaling has been described in various tumor entities as a central modulator of the tumor microenvironment (summarized in **Figure 1** and **Table 1**). To date, only limited direct data are available for urothelial carcinoma. However, initial analyses indicate a possible relevance. Whether the mechanisms described in other tumor types are also effective in UC has not yet been systematically investigated. The current evidence for the role of the CS and, more specifically, C5a receptors in UC is presented below.

**Table 1:** Systematic overview of C5aR1- and C5aR2-specific studies across cancer entities: experimental models, biological effects, tumor-promoting or tumor-suppressing activity, and translational relevance.

Receptor	Entity	Study model	Effect	Pro-tumoral	Anti-tumoral	Level of evidence	Translational relevance	Reference
C5aR1	Lung cancer	Murine model, murine xenograft model	Recruitment of MDSCs resulting in limited CD8^+^ T-cell response	x		preclinical	low to intermediate	(31, 36)
	Breast cancer	Murine model	Promotion lung metastasis by inducing TGF-β and IL-10 production by immature myeloid cells and altering the T-cell response	x		preclinical	low	(28)
	Ovarian cancer	Human samples (blood, ascites)	Receptor activation on neutrophils results in suppressed T-cell response	x		translational	intermediate to high	(32, 33)
	Hepatic metastasis	Murine model	Attraction of MDSCs und suppression of effector T-cell responses	x		preclinical	low	(35)
	Lung cancer metastasis	Murine model	Amplification of tumor-supporting activity of neutrophil MDSCs *via* the formation NETs	x		preclinical	low	(38)
	Urothelial carcinoma	Patient cohort with tissue samples	5-year survival rate, and the disease-specific 5-year survival rate were lower in C5aR1-positive cells	x		clinical	high	(49)
		Patient cohort	Association between high gene expression of C5aR1 and tumor stages	x		translational	high	(51)
	Gastric cancer	Patient cohort with tissue samples	C5aR1^+^ TAMs exhibited an immunosuppressive property that contributed to impaired CD8^+^ T-cell function and serves as a marker for a poorer prognosis	x		clinical	high	(53)
C5aR2	Melanoma	Murine model	Counteracts tumor development		x	preclinical	low	(37)
	Colorectal cancer	Murine model	Tumor growth restriction		x	preclinical	low	(40)
	Cutaneous squamous cell carcinoma	Murine model	Tumor growth restriction		x	preclinical	low	(41)
	Glioma	Patient cohort with tissue samples	Correlation between upregulated and a poor prognosis, which is also associated with the expression of PD-L1, PD-1, TIM-3, CTLA-4, and Siglec-15, as well as markers of TAMs, Th2 cells, and Treg cells	x		clinical	high	(42)
	Breast cancer	Patient cohort with tissue samples	High expression of the receptor is related to poor prognosis	x		clinical	high	(39)
		Murine xenograft model	CD10^+^C5aR2^+^ CAFs are associated with poor survival rates and support CSCs → promotion of tumor growth and chemoresistance	x		preclinical	intermediate	(43)
	Lung cancer	Murine xenograft model	CD10^+^C5aR2^+^ CAFs are associated with poor survival rates and support CSCs → promotion of tumor growth and chemoresistance level	x		preclinical	intermediate	(43)

Level of evidence was categorized according to study design as preclinical, translational, or clinical. Translational relevance was classified as low, intermediate, or high based on proximity to patient application.

## The complement system in urothelial carcinomas

Regarding the role of the CS in UCs, it has been shown that patients with advanced-stage tumors of UTUC and BC had significantly elevated levels of C1s, which were associated with adverse clinicopathological parameters. Also C1s was shown to be an independent poor prognostic factor (44). The transmembrane protein CD46 is known to be a co-factor in degrading complement components and thus regulating complement activation (45, 46). Matrix metalloprotease 9 (MMP9) expression was positively influenced by CD46 alteration in BC cell lines. The phosphorylation of protein kinase B (AKT) and p38 mitogen-activated protein kinases (p38 MAPK) triggered by CD46 overexpression resulted in enhanced activator protein 1 (AP-1) activity. Inhibition of the p38 MAPK and AKT pathways lessened the CD46-induced upregulation of AP-1 and MMP9. CD46 induced upregulation of MMP9 resulted in increased migratory and invasive capabilities (47). In a recent proteomic and *in silico* analysis, the complement cascade was highlighted as an enriched pathway in BC. C5, along with other proteins of the CS, showed significant correlations with all immune cell infiltrations (48).

### C5a receptors in urothelial carcinomas

As described above, C5aR1 and C5aR2 can play a pro- or anti-tumoral role in various cancer entities. The role of C5aR2 in UCs has not been described in the literature to date, whereas the role of C5aR1 has been explored to a limited extent. A study investigating the role of C5aR1 in urothelial carcinoma after radical surgery (cystectomies and nephroureterectomies) was conducted by Wada et al. in 2016. The authors were able to detect C5aR1 expression in nearly three-quarters of patients. It was also demonstrated that the overall survival rate, the 5-year survival rate, and the disease-specific 5-year survival rate were lower in patients with C5aR1-positive cells, leading the authors to conclude that C5aR1 expression is associated with a poorer prognosis in UC patients (49) (**Figure 1** and **Table 1**). A subsequent meta-analysis by Wang et al. (50) on the prognostic significance of C5aR1 in human solid tumors supports these findings. A recent pan-cancer analysis revealed that the expression of C5, as well as C5aR1, was downregulated in human BC compared to adjacent normal tissue. The analysis of differential gene expression at different tumor stages also showed an association between high gene expression of C5aR1 and tumor stages (**Table 1**) (51). The authors deduced from their research that C5aR1 could be a possible biomarker for cancer diagnosis, prognosis, and treatment outcomes, and could therefore be a potential target for immunotherapies (51). Clinical application of C5aR1 as a prognostic biomarker is not yet possible, since further studies are still needed to address aspects such as reproducibility across independent patient cohorts, standardization of immunohistochemical assessment, intratumoral heterogeneity, and the distinction between predictive and prognostic utility. There is also a need for studies comparing C5aR1 with biomarkers currently established for UC to eventually establish its clinical application.

## C5a receptor as a potential therapeutic target in urothelial carcinoma

Several studies have already shown that C5aR1 signaling is a valuable target for cancer therapy, especially in combination with existing treatment options, such as chemotherapy or immune checkpoint inhibitors. C5a promoted pro-tumorigenic effects in the K14-HPV16 transgenic mouse model for squamous cell carcinogenesis by suppressing the cytotoxicity of CD8^+^ T cells. Inhibition of C5a/C5aR1 signaling by the specific antagonist PMX53 in combination with paclitaxel chemotherapy enhanced the activity of CXCR3^+^ effector memory CD8^+^ T cells (52). Another research group showed that in gastric cancer, C5aR1^+^ TAMs exhibited an immunosuppressive property that contributed to impaired CD8^+^ T cell function and served as a marker for a poorer prognosis. This immunosuppressive function of TAMs was reversed by blocking C5aR1, which resulted in the reactivation of anti-tumor immunity by CD8^+^ T cells and promoted the response to anti-PD-1 therapy (53). In high-grade serous ovarian cancer, C5aR1 is mainly expressed by TAMs and is associated with an immunosuppressive TME and an unfavorable disease course. The tumors are also characterized by a moderate response to anti-PD-1/PD-L1 therapy and dense infiltration by TAMs and Treg cells. Blockade of C5aR1 enhanced the antitumor efficacy of anti-PD-1 immunotherapy in preclinical models (54). These data led to the development of clinical trials combining PD-1/PD-L1 blockade with C5a/C5aR1 inhibition. In a Phase I clinical study the combination of an anti-C5aR1 monoclonal antibody (avdoralimab) with duravalumab (anti-PD-L1) was tested in patients with advanced hepatocellular carcinoma, UC, renal cell carcinoma, or NSCLC, where the patients had received at least one systemic therapy (NCT03665129) (55, 56). However, despite initial positive results in lung and liver cancer, the trial was suspended because the combination of synergistic C5aR1 and PD-L1 blockade only showed minimal anti-tumor activity in an extension study (56). A Phase I clinical study was also conducted to assess the safety, tolerability, and pharmacokinetics of TJ210001 (monoclonal anti-C5aR1 antibody) in patients with refractory or recurrent advanced solid tumors (NCT04678921) (57). As far as we know, no results have been published yet. A Phase I dose escalation study with TJ210001 in patients with advanced solid tumors is also ongoing (NCT04947033) (57). Recent studies have shown that the anti-tumor efficacy of radiation therapy can be positively influenced by specifically targeting the complement system. Mouse models for NSCLC and colorectal cancer showed that the combination of radiation therapy and C5aR1 blockade revived the anti-tumor immune cell response and improved survival rates (58, 59). From a clinical perspective, the described findings provide a compelling rationale for their inclusion in modern treatment strategies for UC. Patients with high-risk NMIBC receive intravesical installations of Bacillus Calmette-Guérin (BCG) as the standard of treatment, but a substantial fraction of patients do not respond to BCG treatment (60, 61). Studies investigating the BCG failure mechanisms have shown that a TME with high concentrations of CD25^+^ Treg cells and TAMs prior to treatment was a predictor of poorer recurrence-free survival following BCG therapy (62), and that the TME of non-responders was rich in exhausted CD8^+^ PD-L1^+^ T cells (63). Based on the studies mentioned above and these findings, it can be speculated that specifically targeting the C5a/C5aR1 axis could therefore prove to be a novel strategy for overcoming BCG resistance. However, direct evidence supporting the application in UC are still pending and therefore specific studies need to be undertaken. In advanced and metastatic UC, immunotherapies have made considerable progress (64). Acquired resistance to immunotherapy represents a major challenge in solid tumors and limits long-term success in up to 65% of patients who respond initially to immunotherapy (65). One mechanism underlying non-response is that many solid tumors, including UC, employ strategies to evade recognition and elimination by the immune system, for example, by suppressing the anti-tumor T-cell response via PD-1 and PD-L1 (66, 67). C5a/C5aR1 blockade could potentially overcome resistance to immunotherapy in this patient group. It should be noted, nevertheless, that the NCT03665129 trial was discontinued because the combination of C5aR1 and PD-L1 blockade showed only a minimal antitumor effect (56). Therefore, to overcome resistance to immunotherapies, other targets for combination therapy must be identified. However, the C5a/C5aR1 axis appears to be a promising approach for future UC-specific studies that investigate complement inhibition in combination with guideline-based therapies.

## Conclusion

Both anaphylatoxin receptors C5aR1 and C5aR2 have been shown to feature important functions in the pro- or anti-tumor responses in various cancer entities. In UCs specifically, C5aR1 expression appears to act as a prognostic marker and could be a potential target for immunotherapies, possibly in combination with already known therapies. There have been no reports of C5aR2 in UCs to date. This reinforces the fact that the role of C5aR2, together with C5aR1, especially in UCs still needs to be studied in great depth in order to develop tailored therapy options that consider the individual immune response of the patient. Future studies should prioritize the following areas: (i) Large-scale cohort studies to validate C5aR1 as a prognostic and potentially predictive biomarker, as well as to determine C5aR2 expression patterns. It should be noted that the most recent studies on UC identify molecular subtypes (luminal papillary, luminal-nonspecified, luminal-unstable, stroma-rich, basal/squamous and neuroendocrine-like) that may influence immune infiltration, response to therapy, and prognosis in individual patients (reviewed in 68). Therefore, it is critical in cohort studies, therefore, it is critical to ensure that all subtypes are either included or to take these subtypes into account during analysis and to examine whether complement signaling differs among the known subtypes. Recent advances in single-cell transcriptomics, spatial transcriptomics, multiplex imaging, and spatial proteomics have significantly improved our understanding of immune microenvironments and are therefore of particular importance for the study of complement-mediated cellular interactions. Consequently, these technologies should be applied in future studies of UCs. (ii) Mechanistic studies using UC cell lines and mouse models to characterize the biological roles of both receptors. These studies are the first steps toward determining whether it would be possible to incorporate complement-targeted therapies into UC treatment regimens in the future.
